# An Analytical Description of Certain Stony Concretions (Phosphates of Lime), Coughed up from the Lungs by Joseph Shildigger, a Patient in the New-York Hospital, with Practical Remarks on Their Formation

**Published:** 1803-10-01

**Authors:** M. Le Baron de Carendeffez


An ANALYTICAL DESCRIPTION of certain STONY CONCRETIONS *(Phosphates of Lime), coughed up from the Lungs by* JOSEPH SHILDIGGER, *a Patient in the New-York Hospital, with Practical Remarks on their Formation. By M.* LE BARON DE CARENDEFFEZ. *Read before the Physical Society of New-York, October* 6, 1802.

THIS man was by trade a stone-cutter, and was admitted into the hospital with phthisical symptoms. He had great difficulty in breathing, violent cough, expectoration of pituitous and purulent matter, sometimes mingled with blood, considerable emaciation and night sweats. He also frequently brought up small stones, which have amounted, by estimation, to more than two hundred. In proportion as these have been voided, his symptoms have been relieved. A suspicion had arisen that these stony concretions were formed from the dust, inhaled while he was at work in shaping quarry-stones, by his mallet and chisel, for the purposes of architecture. A parcel of these hard substances which he had spit up, having been given by the patient to the attending physician, and obligingly put into my hands for examination by Dr. Mitchill, I take this opportunity of communicating to the Society the result of my inquiries.

These morbid or preternatural calculi were of irregular shape, very hard, and of a greyish or pale slate colour.

When subjected to the operation of acids, both vegetable and mineral, for some time they became white, and seemed to be softer as they lost their grey colour, which appeared to be derived from a glutinous slime.

Treated by watery solutions of alkalies, both in their caustic and carbonated states, this glutinous substance was not decomposed, but separated, dissolved, and converted into a whitish compound resembling soap.

Some of the concretions were then mixed with an equal weight of carbonate of soda, grown dry by spontaneous efflorescence, and exposed to the heat of a reverberatory furnace, to determine whether they were of a siliceous nature. But nothing of a vitrifiable nature was observed. Failing thus to obtain any kind of glass, I concluded they contained no siliceous ingredient. The crucible then being taken from the fire, cooled and inspected, I was surprised to find the concretions in a heap, and suspended, as it were, in my crucible, without having undergone any change of their original figure, and without manifesting the smallest signs of vitrification. At the same time the whole inside of the bottom of the vessel was turned to glass, by means of the siliceous matter, which, as is well known, is a constituent ingredient of the Hessian crucibles which I employed.

On taking them from the crucible I found they had become brittle, and could easily be rubbed to pieces between the fingers: the gluten had been consumed, and they had become as white as in the former experiments.

On weighing them again, they were found to have lost scarcely three grains of their original weight; a loss which I ascribe to the animal gluten which connects, and, as it were, cements the particles together.

Some of the calcined matter being then reduced in a mortar to a very fine powder, I first diluted it with a little water, and afterwards poured on it about two-fifths of its weight of good sulphuric acid. Having been mixed well, they were exposed for three hours to a moderate sand-head. On examining the mass, I found it thick, viscous, and adhesive. I diluted it with water, and it reddened the tincture of litmus, and possessed a sour but agreeable taste. This was easily distinguishable for being an acidulous phosphate of lime: 1. By its dissolubility in water; 2. By its precipitating with lime-water, a regenerated insoluble calcareous phosphate, which was not decomposable by alkalies; 3. By its forming, with ammoniac, soda, pot-ash, and magnesia, as many particular phosphates. These phosphates precipitated a true phosphate of lime from the mixture, by depriving it of the surplusage of acidity; and from this surplusage only these new phosphates were formed. To this excess of phosphoric acid, the mass owed its solubility bility and fluidity, for as soon as it lost this superfluous portion, it became an insoluble precipitate.

The oxalic acid acted upon this acidulous phosphate in a much more powerful manner. It decomposed it entirely by attracting its calcareous basis. Therewith it formed immediately an insoluble oxalate of lime. This fell to the bottom, while the liberated phosphoric acid swam in the liquid above it. I washed this precipitated oxalate of lime in water until it became tasteless, and then, on decomposing it by the carbonates of potash and soda, obtained carbonates of lime.

The sulphate of lime, formed by a decomposition of a part of these concretions, was purified and separated from the acidulous phosphate of lime by repeated washings. Being unable to decompose this earthy salt by carbonates of potash, soda, or ammoniac, I succeeded in forming an hydro-sulphure of lime by means of powdered charcoal in a sufficient heat. From this I obtained the sulphur by means of vinegar.

The result of all these experiments is, *that these pulmonary stones or concretions are true* PHOSPHATES OF LIME.

Their formation is owing probably to the great quantity of this calcareous salt carried into the system, with both vegetable and animal food. In order to keep it dissolved in the fluids, the constitution ought to be supplied with a surplusage of phosphoric acid. When there is a deficiency of this phosphoric menstruum, these concretions are formed in different parts of the body. Hence, when there is no excess of phosphoric acid in the blood and secretions, we so often find concretions similar to these in the kidneys, in the bladder, in the bronchia, in the lungs, and in other places.

There is every reason to believe, both from the probability of the thing, and from chemical experiments, that such concretions as these would not be formed, if there existed the requisite superabundance of phosphoric acid. For if this was present in sufficient quantity, it would soften, dissolve, and hold in solution the neutral earthy salt in all cases, after the same manner that the great quantity of it in healthy urine is kept dissolved and suspended,

*The oxalic and sulphuric acids seem to have a powerful agency in totally loosening the compages of bones, and of disposing them to be dissolved in water;* while the other acids, of whatever kind, though they may appear to dissolve them, do no more in fact than separate the particles, which which, instead of undergoing solution, are precipitated in the form of a white and granulated powder.

All these facts which I have seen and derived from my own experience, in submitting these concretions to the action of different acids, and all the others which I have gathered from experiments made on calculi of the kidneys and bladder, convince me that most reliance is to be placed on THE OXALIC and PHOSPHORIC ACIDS for destroying these terrible concretions. While the *nitric* and *muriatic* acids recommended by Messrs. Fourcroy and Vauquelin, do not act so powerfully upon these calculi, are more disagreeable to the taste, and are more stimulant upon the living parts, without having a proportional action upon the stones. On the other hand, considering that the *oxalic* and *phosphoric* acids may be exhibited in greater quantity and higher concentration than the others, I think them highly deserving the attention of physicians: I therefore recommend them to their notice and trial, as promising to do much in the cause of humanity, both in the form of drinks and injections.

